# Digital rectal examination and its associated factors in the early detection of prostate cancer: a cross-sectional population-based study

**DOI:** 10.1186/s12889-019-7946-z

**Published:** 2019-11-27

**Authors:** Samara Carollyne Mafra Soares, Marianna de Camargo Cancela, Arn Migowski, Dyego Leandro Bezerra de Souza

**Affiliations:** 1 Graduate Program in Collective Health, Federal University of Rio Grande do Norte, Natal, Rio Grande do Norte Brazil; 2 Population Research Division, Brazilian National Cancer Institute (INCA), Rio de Janeiro, Brazil; 3 Cancer Early Detection Division, Brazilian National Cancer Institute (INCA), National Institute of Cardiology (INC), Rio de Janeiro, Brazil; 4 Collective Health Department, Federal University of Rio Grande do Norte, Natal, Rio Grande do Norte Brazil; 5 Present Address: Universidade Federal do Rio Grande do Norte / Federal University of Rio Grande do Norte. Programa de Pós-Graduação em Saúde Coletiva / Graduate Program in Collective Health, Avenida Senador Salgado Filho 1787, CEP: 59010-000 Lagoa Nova, Natal, RN Brazil

**Keywords:** Digital rectal examination, Excessive use of health products and services, Prostate neoplasms, Public Health,mass screening; Cancer early detection

## Abstract

**Background:**

Digital rectal examination (DRE) is one of the most common strategies for prostate cancer early detection. However, the use for screening purposes has a controversial benefit and potential harms can occur due to false-positive results, overdiagnosis and overtreatment. The objective of this study is to calculate the prevalence and identify factors associated with the receipt of DRE in Brazilian men.

**Methods:**

We selected men older than 40 from a nationwide population-based survey (13,625 individuals) excluding those with prostate cancer diagnosis. Information was extracted from the most recent database of the Brazilian National Health Survey (PNS 2013). Statistical analysis was carried out to calculate incidence rate ratios, with 95% confidence intervals and *p* values, through multivariate analysis with Poisson regression and robust variance.

**Results:**

Men having private health insurance (63.3%; CI = 60.5–66.0) presented higher prevalence of DRE than those in the public health system (41.6%; CI = 39.8–43.4). The results show a positive association between DRE and men having private health insurance, aged 60–69, living with a spouse, never smokers, and living in urban areas. Among public health services users, this positive association was observed among men aged 70–79, living with a spouse, having bad/very bad health self-perception, abstainers, ex-smokers, with undergraduate studies, presenting four or more comorbidities, and residing in urban areas.

**Conclusions:**

Prostate cancer screening with DRE is quite frequent in Brazil, specially among men with private health plans and better access to health services, healthier lifestyle and at more advanced ages, characteristics which increase the risk of overdiagnosis and overtreatment.

## Background

Brazil is the fifth most populous country in the world, with over 209 million inhabitants. The cancer estimates for 2018–2019 indicate prostate cancer as the most incident type of cancer in Brazilian men (31.7%), aside from non-melanoma skin cancer, [1]. One of the likely explanations for this high incidence is overdiagnosis due to the widespread of prostate cancer screening [2].

In Brazil there is a very strong annual campaign promoting prostastate cancer screening with digital rectal examination (DRE). Many of the campaigns promoting screening with DRE have been around in the country since the 1990s, and by 2007 screening for prostate cancer with DRE was almost unanimous even in the context of universities where there is usually greater access to up-to-date information, with research showing that among medical students showed that 90.9% recommended routine prostate cancer screening with DRE [3]. To further strengthen the practice of screening with DRE in the country, in 2008 was created the most powerful of these campaigns with the name “One touch, a dribble”, making reference to DRE and football/soccer, because in Brazil DRE is called “rectal touch” and the word “touch” is also used in soccer. In the last campaign (2018) the leaflet for general population said that “Even in the absence of symptoms, men from the 50 years, or 45 if there is a family history or black race, should go to the urologist annually to perform rectal touch and take the blood PSA test”. Next, the leaflet emphasizes the importance of screening for prostate cancer with DRE: “No symptoms not warrant that there are no problems. Therefore, take preventive exams” and “20% of prostate cancer patients are diagnosticated only by changes in rectal touch” [4]. To this day there are countless media campaigns advocating prostate screening with DRE on Brazil, including internet sites, outdoors and leaflets for the general population.

Urologists are generally regarded as the main media spokespersons on prostate cancer screening and are the main opinion makers on this issue among the general population and other health professionals in Brazil [5]. A study of the focus of the major Brazilian written media (largest newspapers and magazines in the country) with data up to 2016, that only 7% of these reports were opposed to prostate cancer screening. This study showed that of the reports that mentioned the recommended screening tests, 86.5% recommended screening with DRE, 77% mentioned screening with PSA and DRE, 9.5% only DRE and 13.5% mentioned only screening with PSA [5].

In the Brazilian scenario for health services, the access to prostate cancer screening examinations is provided by the Brazilian Unified Health System (Brazil’s publicly funded national health system, SUS), created in 1988. SUS provides universal and free access to the entire population. There are also private health insurances, also kwnown as supplementary health, which provide additional healthcare to 25% of the population [6].

In Brazil, in supplementary health, routine medical appointments are usually made directly with a urologist, and in the SUS, routine consultations for prostate cancer screening are carried out by GPs, geriatricians or urologists and the recommendation of the Brazilian Society of Urology is that men should seek a urologist once a year for annual screening with DRE and PSA beginig with 50 years or 45 years for afro-descendents or men with family history of prostate cancer [7]. This recommendation is also supported by some influential international guidelines to date, such as the current National Comprehensive Cancer Network guidelines [8]. In a study published in 2018 in which doctors from different regions of Brazil answered questions about the their screening practice, 92.6% of the urologists recommended routine screening for prostate cancer [9]. In this study 78.8% of the urologists, 32% of geriatricians, and 20.1% of the GPs recommended routine screening with DRE. The same study shows that most GPs (82.6%) and geriatricians (69.5%) are also in favor of routine prostate cancer screening, but prefer to screen with PSA alone, and it is important to consider that in Brazil among these doctors it is common the practice of referring patients to a urologist to permorm DRE screening [9].

Despite the importance of early diagnosis in people that present the initial signs and symptoms of the disease, screening of asymptomatic individuals via PSA and/or DRE is extremely controversial. This occurs in function of arguments related to its efficacy and existence of harms associated with false-positive results, overdiagnosis and overtreatment [10, 11]. For DRE screening there are no conclusive evidences on its efficacy and for PSA, there are controversies [12].

Even considering the harms associated with prostate cancer screeening, the lack of evidence on efficacy of screening with DRE and the widespread of prostate cancer screening with DRE in Brazil, there is little information regarding this practice in the country [13]. Nowadays, in face of the new guidelines against screening, it is paramount to verify whether medical practice is aligned with such recommendations, for public and private healthcare systems. The objective of this study is to establish the prevalence of DRE and its associated factors, in the male Brazilian population, over 40 years of age, and identify the differences between public and private healthcare practices.

## Methods

An epidemiological cross-sectional study is presented herein, utilizing data from the most recent version of the Brazilian National Health Survey (PNS), a transversal representative survey of the entire adult Brazilian population, carried out in 2013 by the Health Ministry and the Brazilian Institute of Geography and Statistics (IBGE) [14, 15]. There is no plan to include the question about DRE in the next issues of PNS, so the study of this base is a unique opportunity to know the prevalence of prostate cancer screening with DRE in Brazil.

The sampling of PNS was household-based, with conglomerate stratification in three stages: census sectors (primary units); families (second step) and adult inhabitants (third step) [15, 16].

A total of 81,254 households were visited, and 64,308 individuals were selected over the age of 18. Finally, 60,202 interviews were carried out, encompassing 93.6% of the sample, with a refusal rate of 2.7% (1717 adults) and absence rate 3.7% (2389) [16].

The study selected all men over the age of 40 who participated in PNS, and those who presented prostate cancer diagnosis were excluded from the study (totaling 13,625 individuals). Previous history of cancer leads to DRE for monitoring purposes, which would be a confounding variable for the objective of this study. Prevalence of Digital Rectal Examination (DRE) with screening purposes was calculated through the question: “When was the last time you had a physical/digital rectal examination of the prostate?”, with the answers categorized in yes or no, without consideration of the time elapsed since the most recent DRE. The answers to the question leave no doubts regarding the possibility of undergoing DRE for reasons (e.g., pathologies and other issues) other than routine tests for early detection of prostate cancer and is followed by another question about the reasons why the man never actively sought a DRE, such as prejudice and other known barriers for prostate cancer screening with DRE. As the objective of the study is to identify differences between public and private healthcare practices, analysis were stratified: DRE in private practice and DRE in SUS, the Unified Public Health System. Estimates of prevalences (%) and the respective confidence intervals (95%) were calculated according to the following independent variables: age group (40–49; 50–59; 60–69; 70–79; and over 80 years of age), self-reported skin color (white, non-white), marital status (no spouse, with spouse), health self-perception (very good/good; regular; bad/very bad), consumption of alcohol (abstinent; moderate; excessive), consumption of tobacco (never smoked; ex-smoker; smoker), economic conditions according to the “Economic Classification Criterion Brazil 2010 (CCEB)” (A and B; C; D; E), education level (illiterate, fundamental education, high school, undergraduate studies), employment condition (employed, unemployed), multimorbidity (0 or 1; 2; 3; 4 or more chronic diseases), Brazilian geographic regions (Southeast; South; Midwest; Northeast; North), and type of housing area (urban; rural). A supplementary table was created using the PNS data for characterization between private and public healthcare consumers mainly focusing on their demographic and socioeconomic variables (Additional file 1: Table S1).

CCEB 2010 enables classification in social strata through an estimate of the purchasing power of individuals [17].

Multimorbidity was defined as the presence of two or more chronic diseases simultaneously in the same individual [18]. The amount of co-existent chronic diseases was utilized as a category, associated with the degree of multimorbidity.

Statistical software Stata® 14 was utilized for tabulation and statistical analysis, with a command for complex samples, verifying the prevalence ratios (PR) with 95% confidence intervals (CI 95%) and *p* values(≤0.05). Multivariate analysis utilized Poisson Regression with robust variance.

## Results

Prevalence of DRE among men that utilized private health insurance-related services was 63.3% (CI = 60.5–66.0) and among men that utilized the publicly funded healthcare system was 41.6% (CI = 39.8–43.3). Descriptive analysis showed that for all variables and all categories, the prevalences were higher for the group containing private healthcare users (Table 1). The proportion of men in the three highest age groups (60–69; 70–79; and over 80 years of age) and the time elapsed since the most recent examination is very similar for all moments analyzed. Approximately 21.6% of men aged 40–49 years old, 37.5% of those aged between 50 and 59 years old and 45.3% of men aged between 60 and 69 years old had undergone DRE in the last two years (Table 2).

**Table 1:** Prevalence (%), respective confidence intervals and estimate of the population n from the complex sample of Digital Rectal Examinations in Brazilian men, with private health plan and SUS users, according to the independent variables

	Private		Public	
Variables	Prev (%) CI (95%)	n estimated pop	Prev (%) CI (95%)	n estimated pop
Age group				
40–49	38.1 (33.8–42.7)	1.405.763	23.2 (20.7–26.0)	1.948.036
50–59	66.5 (60.9–71.6)	2.460.502	44.2 (40.9–47.6)	3.368.704
60–69	86.1 (81.8–89.5)	1.632.061	56.3 (52.3–60.3)	2.597.727
70–79	87.2 (80.9–91.6)	883.170,10	63.5 (57.9–68.7)	1.597.197
80	87.1 (70.7–94.9)	486.952,40	53.7 (44.7–62.4)	493.567,30
Self-reported skin color				
White	64.8 (61.3–68.1)	4.562.037	44.9 (42.0–47.8)	4.714.661
Non white	60.5 (55.8–65.0)	2.306.411	39.0 (36.8–41.4)	5.290.570
Marital status				
No spouse	56.1 (50.3–61.7)	935.843,30	34.6 (31.7–37.7)	2.127.869
With spouse	64.6 (61.4–67.6)	5.932.605	44.0 (42.0–46.1)	7.877.363
Health self-perception				
Very good/good	61.2 (57.9–64.5)	4.865.342	35.8 (33.5–38.2)	4.485.569
Regular	68.5 (62.5–73.8)	1.752.565	47.1 (44.1–50.0)	4.359.475
Bad/Very bad	71.6 (55.6–83.5)	250.542,30	51.0 (46.1–55.8)	1.160.187
Consumption of alcohol				
Abstinent	63.8 (60.2–67.3)	4.286.342	44.3 (42.2–46.4)	7.433.818
Moderate	63.4 (58.4–68.2)	2.077.424	39.2 (35.4–43.1)	1.924.645
Excessive	58.2 (48.8–67.1)	504.683,40	27.4 (23.1–32.3)	646.768,60
Consumption of tobacco				
Never smoked	61.5 (57.9–65.0)	3.693.281	41.3 (38.8–44.0)	4.518.163
Ex-smoker	72.0 (66.9–76.5)	2.392.354	49.4 (46.2–52.6)	3.687.023
Smoker	51.2 (43.2–59.1)	782.812,70	31.8 (28.5–35.3)	1.800.046
Brazil Criterion				
A and B	67.1 (62.3–71.5)	2.744.026	44.0 (39.3–48.9)	1.937.959
C	60.4 (55.6–65.0)	2.333.540	43.7 (40.8–46.6)	4.170.152
D and E	61.8 (56.7–66.5)	1.790.882	38.8 (36.3–40.9)	3.897.120
Education level				
Iliterate	70.2 (57.9–80.2)	273.077,60	38.3 (34.7–42.1)	1.700.334
Primary	65.4 (60.1–70.4)	2.152.602	41.9 (39.6–44.3)	5.804.146
High school	57.0 (52.1–61.8)	1.944.312	41.6 (37.4–45.9)	1.864.492
Undergraduate	66.3 (61.3–71.0)	2.498.457	49.1 (42.3–56.0)	636,258,9
Employment				
Employed	57.7 (54.3–61.1)	4.442.582	36.1 (34.0–38.2)	5.552.262
Unemployed	76.7 (71.2–81.5)	2.425.866	51.4 (48.3–54.5)	4.452.969
Multmorbidity				
0 or 1	57.9 (54.6–61.2)	4.203.414	35.2 (33.2–37.3)	6.109.789
2	72.5 (65.3–78.6)	1.334.487	53.2 (48.8–57.5)	2.001.653
3	77.3 (68.4–84.3)	786.950,30	61.7 (55.1–67.8)	1.085.068
4 or more	73.5 (62.9–81.9)	543.597,40	68.3 (60.7–75.1)	808.721,10
Geographic region				
Southeast	63.7 (59.5–67.7)	3.985.687	46.4 (43.1–49.9)	4.446.176
South	66.2 (60.1–71.8)	1.252.068	41.1 (36.7–45.7)	1.452.174
Midwest	62.9 (57.1–68.3)	512.759,40	45.5 (41.4–49.6)	775.189,50
Northeast	60.9 (54.8–66.6)	926.761,90	37.1 (34.2–40.0)	2.715.986
North	52.5 (42.8–62.0)	191.171,70	32.1 (28.6–35.8)	615.705,50
Housing area				
Urban	64.2 (61.3–67.0)	6.707.967	43.6 (41.4–45.7)	8.230.541
Rural	39.1 (30.1–48.9)	160.481,90	34.4 (31.7–37.1)	1.774.691

**Table 2:** Proportion of men that underwent DRE, according to age groups and time elapsed since last examination

Last examination	Less than one year	Less than two years and more than 1 year	Between two and three years	Over three years ago	Never
Age group	Prop (%) CI(%)	Prop (%) CI(%)	Prop (%) CI(%)	Prop (%) CI(%)	Prop (%) CI(%)
40–49	11.9(10.1–13.9)	9.1(7.7–10.6)	3.6(2.8–4.6)	3.2(2.6–4.0)	72.2(69.9–74.5)
50–59	22.3(19.8–25.0)	15.2(13.3–17.3)	6.0(4.9–7.4)	8.0(6.5–9.8)	48.5(45.6–51.4)
60–69	30.2(27.0–33.5)	15.1(12.9–17.5)	7.4(5.8–9.3)	12.4(10.4–14.7)	35.0(31.8–38.4)
70–79	26.0(22.0–30.4)	14.0(11.1–17.5)	6.6(4.7–9.2)	23.7(19.6–28.4)	29.8(25.7–34.2)
80 or more	21.4(15.8–28.4)	13.1(8.4–19.7)	5.1(3.0–8.6)	26.7(21.0–33.3)	33.7(26.7–41.6)
Total	20.5(19.2–21.9)	12.8(12.0–13.9)	5.5(4.9–6.1)	9.6(8.6–10.6)	51.7(50.1–53.3)

Bivariate analysis of DRE in private health insurance users showed that DRE presented a very similar PR in the three highest age groups, being higher in the age group 70–79 years old (PR = 1.31; CI = 1.19–1.45), and over 80 years old (PR = 1.31; CI = 1.12–1.54). This analysis showed that for both types of users (private health insurance and public healthcare system) there was a statistically significant positive association between DRE and the age group 70–79 years old (PR = 1.31; PR = 1.43; respectively, for private and public users), in ex-smokers (PR = 1.40; PR = 1.56), in men with three or more chronic diseases (PR = 1.34; PR = 1.94), men who reported health condition as bad/very bad (PR = 1.17; PR = 1.42), unemployed (PR = 1.33; PR = 1.43), living with a spouse (PR = 1.15; PR = 1.27) and inhabitants of urban areas (PR = 1.64; PR = 1.27) (Table 3) .

**Table 3:** Bivariate and multivariate analyses of Digital Rectal Examination in Brazilian men, private health plan and SUS users, according to the independent variables

	Private				Public			
Variables	PR CI (95%)	*p* value	Adjusted PR CI (95%)	p* value	PR CI (95%)	p* value	Adjusted PR CI (95%)	p* value
Age group								
40–49	0.57(0.50–0.66)	< 0.001	0.57(0.50–0.66)	< 0.001	0.52(0.46–0.60)	< 0.001	0.54(0.47–0.62)	< 0.001
50–59	1		1		1		1	
60–69	1.30(1.19–1.41)		1.32(1.21–1.43)		1.27(1.15–1.41)		1.22(1.10–1.35)	
70–79	1.31(1.19–1.45)		1.31(1.19–1.45)		1.43(1.28–1.60)		1.34(1.20–1.49)	
80	1.31(1.12–1.54)		1.30(1.12–1.52)		1.21(1.01–1.46)		1.31(1.09–1.57)	
Self-reported skin color								
White	1	0.149	–	–	1	< 0.001	–	–
Non white	0.93(0.85–1.02)		–	–	0.87(0.80–0.95)		–	–
Marital status								
No spouse	1	0.014	1	0.006	1	< 0.001	1	< 0.001
With spouse	1.15(1.03–1.29)		1.15(1.04–1.26)		1.27(1.15–1.40)		1.26(1.15–1.38)	
Health self-perception								
Very good/good	1	0.043	–	–	1	< 0.001	1	0.009
Regular	1.12(1.01–1.23)		–	–	1.31(1.20–1.43)		1.13(1.04–1.23)	
Bad/Very bad	1.17(0.95–1.44)		–	–	1.42(1.27–1.60)		1.25(1.11–1.42)	
Alcohol consumption								
Abstinent	1.10(0.93–1.29)	0.548	–	–	1.61(1.35–1.92)	< 0.001	1.29(1.09–1.54)	0.016
Moderate	1.09(0.91–1.30)		–	–	1.43(1.18–1.73)		1.26(1.05–1.52)	
Excessive	1		–	–	1		1	
Tobacco consumption								
Never smoked	1.20(1.02–1.42)	< 0.001	1.24(1.07–1.44)	0.014	1.30(1.15–1.47)	< 0.001	1.23(1.10–1.38)	< 0.001
Ex-smoker	1.40(1.18–1.67)		1.20(1.03–1.40)		1.56(1.38–1.76)		1.27(1.13–1.42)	
Smoker	1		1		1		1	
Brazil criterion								
A and B	1	0.107	–	–	1	0.016	–	
C	0.90(0.81–1.00)		–	–	0.99(0.88–1.12)		–	
D and E	0.92(0.83–1.02)		–	–	0.88(0.77–0.99)		–	
Education level								
Iliterate	1	0.019	–	–	1	0.044	1	< 0.001
Primary	0.93(0.78–1.11)		–	–	1.09(0.98–1.22)		1.17(1.05–1.31)	
High school	0.81(0.68–0.97)		–	–	1.09(0.94–1.25)		1.35(1.18–1.55)	
Undergraduate	0.94(0.79–1.13)		–	–	1.28(1.08–1.52)		1.67(1.42–1.97)	
Employment								
Employed	1	< 0.001	–		1	< 0.001	–	
Unemployed	1.33(1.21–1.46)		–		1.43(1.32–1.54)		–	
Multimorbidity								
0 or 1	1	< 0.001	–		1	< 0.001	1	< 0.001
2	1.25(1.13–1.38)		–		1.51(1.37–1.67)		1.24(1.13–1.37)	
3	1.34(1.19–1.50)		–		1.75(1.55–1.98)		1.32(1.16–1.51)	
4 or more	1.27(1.10–1.47)		–		1.94(1.72–2.19)		1.38(1.23–1.56)	
Geographic region								
Southeast	1	0.247	–		1	< 0.001	1	< 0.001
South	1.04(0.93–1.16)		–		0.89(0.78–1.01)		0.89(0.79–1.01)	
Midwest	0.99(0.88–1.10)		–		0.98(0.87–1.10)		1.06(0.95–1.18)	
Northeast	0.96(0.85–1.07)		–		0.80(0.72–0.89)		0.88(0.79–0.97)	
North	0.82(0.68–1.00)		–		0.69(0.60–0.79)		0.78(0.69–0.89)	
Housing zone								
Urban	1.64(1.28–2.10)	< 0.001	1.68(1.35–2.10)	< 0.001	1.27(1.15–1.39)	< 0.001	1.15(1.05–1.25)	0.004
Rural	1		1		1		1	

When considering bivariate analysis for DRE and men who utilized the public healthcare system, there was also a positive association for men who abstained from alcohol consumption (PR = 1.61), and with higher education levels (undergraduate studies) (PR = 1.28). There was a statistically significant negative association with non-white men (PR = 0.87), belonging to social classes D/E (PR = 0.88), and living in the North region of Brazil (PR = 0.69). There was a higher prevalence ratio in the age group 70–79 years old (PR = 1.43; CI = 1.28–1.60), and for individuals who lived with a spouse (PR = 1.27;CI = 1.15–1.40).

After multivariate analysis for DRE in men who utilized private health plans, the variables that presented statistically significant association (*p* ≤ 0.05) were (Table 3): age group, marital status, consumption of tobacco and housing zone. For the group of men who utilized the public healthcare system, statistically significant association was consolidated for the following variables: age group, marital status, health self-perception, consumption of alcohol, consumption of tobacco, education level, presence of multimorbidities, Brazilian geographic region, and housing zone.

## Discussion

The study presented herein focused on screening of prostate cancer with DRE, through self-reported information by the subjects. The main strength is the representativeness of the population of the fifth most populous country of the world, and to the best of the authors’ knowledge this is the first study to evaluate the prevalence of prostate cancer screening throughout Brazil, and one of the few to evaluate this practice in a low- or middle- income country. Knowledge on this prevalence is important for both the public and private healthcare systems due to the wide dissemination of this type of screening in clinical practice, to the possible harms, and to the high costs associated with it.

The high prevalence of DRE herein verified should be an underestimate of the real magnitude of prostate cancer screening in Brazil. Scientific literature reports that since the end of the 1980’s, DRE started to be complemented or substituted by screening and that after the widespread dissemination of PSA tests, incidence rates for prostate cancer have increased significantly [19, 20]. Therefore, when DRE prevalence data are available, the rates corresponding to PSA blood tests will be the same or higher. Also, there is cultural resistance to adhering to DRE screening in Brazil, which does not occur with PSA [21].

Analysis revealed expressive prevalences for DRE in the male Brazilian population when analyzing individuals that utilized both the private and the public healthcare systems. A population-based study carried out with data of the Multi-Center Health Survey of the state of São Paulo (ISASP) analyzed data on 992 men over the age of 50, between 2001 and 2002. Prevalence of prostate cancer screening was 55.6%, of which 61.8% referred DRE and 73.2% referred PSA [22].

Other studies carried out in Brazil also investigated the prevalence of prostate cancer screening with DRE, but included population coverage limited to a few cities. A study carried out in Juiz de Fora city (Southeast Brazil) analyzed data from 2825 men over the age of 60, and showed DRE prevalence equal to 61.0% and PSA prevalence 75.5% [13]. Another study in Minas Gerais investigated men over the age of 50, all medical doctors that were professors at the medical faculty, and DRE prevalence was 63.7% [23].

For all the age groups in our study, prevalences for users of private health insurances were higher than for those who were assisted by the public health system. This could be in part associated with the different medical practices and approaches of both systems. SUS is based on the primary attention model, involving general practitioners (family doctors), and covering more than 50% of the Brazilian population [24]. The general practitioners within this context are *gatekeepers* regarding the access to specialists. However, in private health insurances is more common to consult directly with a urologist.

Regarding age, it was observed that, in Brazil, DRE became more prevalent in the age groups over 60 years old, compared with the 50–59 years age group. For users of the public healthcare system, prevalence was higher in the age group 70–79 years, and for private health insurance users, higher in the age group 60–69 years. It was also verified that, when considering the date of the most recent examination (less than one year; between one and two years), DRE in men over the age of 80 continues to be carried out in a similar proportion to the younger age groups. This finding is corroborated by other studies that also identified increased prevalences for prostate cancer screening with age, of at least 70 years old, in comparison with younger age groups [19, 20].

Higher occurrence of DRE in older age groups can also be influenced by the prevalences of multimorbidity, as there is a strong statistically significant association between age and multimorbidity, with higher prevalences of multimorbidity in individuals over the age of 60 [18, 25]. Multimorbidity causes higher medical service demands, mainly for primary attention, entailing higher consumption of medicines, higher demands for preventive examinations, higher costs and higher opportunities for screening examination [18, 22], such as DRE and PSA. In Brazil, prostate cancer screening is opportunistic, and the higher the probability of the patient attending health units due to comorbidities, there are higher opportunities for screening referral. It was herein identified that, especially for the services provided by SUS, that the higher the amount of chronic diseases presented by the individual, the higher the prevalence of DRE, pointing to a statistically significant association.

In Brazil in addition to the mass media campaigns that call assintomatic men to go direct to urologists’ offices annually, there is also the practice of geriatricians and GPs to offer screening tests such as DRE and PSA during the consultation for other complains (opportunistic screening). In a recent Brazilian study on the reasons that led patients to seek assistance at primary care, consultations based on suspected warning signs of prostate cancer did not even appear on the exhaustive list of causes cited in the study. In this study only 0.03% of the consultations were classified as urological complaint [26].

The high prevalences of DRE in age groups over 80 years can reveal a cohort effect where people continue to undergo examination, which was a clinical practice very disseminated and stimulated for prostate cancer screening during the last decades. This possibility does not explain the phenomenon observed herein, as DRE prevalence in the last three years in men over 70 years old was above 40%. Screening men over the age of 70, with comorbidities or life expectancy under 15 years, increases the possibility of overdiagnosis and overtreatment, with serious consequences.

When analyzing DRE for the age group 40–49 years, despite this being the age group with lowest prevalence, again prevalence is higher for private health plan users. For this age group, there are only some DRE screening recommendations specific for individuals considered within high risk groups for the disease. For the age group 40–49 years old, there are no evidence of reduction of prostate cancer mortality generated by randomize clinical trials. In this way, these men are being submitted to several harms, with no evidences of the possible benefits.

The socioeconomic conditions verified herein through CCEB, initially suggested an association between better socioeconomic status and DRE, however the association was not confirmed after multivariate stratified analysis per type of service. In a cohort that analyzed the association between socioeconomic status and prostate cancer diagnosis, it was observed that higher socioeconomic conditions were associated with a higher risk of prostate cancer diagnosis, biopsies in younger individuals, and cancers in earlier stages, which was interpreted in function of a more-intense screening behavior and higher medical surveillance in these men [27]. A similar conclusion was obtained in a Finnish randomized study on prostate cancer, which included 72,139 men, where the highest socioeconomic status was associated with overdiagnosis of low-risk prostate cancer and, inversely, with lower risks for incurable cancer and mortality [28]. The hypothesis is that men with higher socioeconomic conditions have better access to health services and screening, leading to early diagnosis, but also to overdiagnosis [29–31].

In the findings presented herein, the education level was positively associated with DRE for the group of men that utilized public health services. No association was verified for private health plan users, probably, due to homogenization regarding this variable within the group. Association between higher education levels and DRE-oriented screening, which was verified herein, was confirmed by other authors [13, 22, 32, 33]. A higher education level is related to a better knowledge of diseases and associated factors, and better notion of self-care, which probably leads to more preventive examinations and screening.

A positive association was confirmed, with statistical significance, between the prevalence of screening examination and marital status “living with spouse”. The study of Santiago et al. (2013) [13] also observed an association between DRE prevalence and marital status “married/living with partner”, suggesting that men with spouses or partners tend to be influenced by them regarding the decision to seek medical attention and undergoing screening examinations.

When analyzing health-related behavior, such as the consumption of tobacco and alcohol, it was observed that the fact of being abstainer or being a social drinker was positively associated with a higher DRE prevalence – but this association was only maintained for men that utilized SUS. Also, non-smokers and ex-smokers underwent more screening examinations, when compared with smokers, and this positive association was maintained statistically significant for both groups (SUS and private health plan users). The consumption of tobacco and alcohol are factors associated with several other illnesses and diseases, and men who refrain from these factors certainly present healthier lifestyles, related to health prevention practices (“*health seeking behavior*”). This phenomenon is well-documented in scientific literature and is one of the causes of overestimation of screening efficacy in several studies, due to the “*healthy screenee bias”*. Men with healthier lifestyles also tend to have lower body mass index and lower risk of prostate cancer, reducing the positive predictive value of DRE, altough high body mass index is associated with a reduction of prostate cancer detection rate during DRE [34].

Another finding herein presented was that men of both analyzed groups (users of public and private healthcare systems) that resided in urban areas underwent more screening examinations for prostate cancer than those residing in rural areas. This geographic difference verified for DRE is probably related to better or worse access to health services and information, in all levels of attention. The sociocultural and economic characterization of these individuals also plays an important role. It is possible that additional factors, present in urban zones, such as proximity to health resources, better access to transportation and more flexible time schedules, can also explain the associations with preventive and screening examinations [27]. Additionally, the men population of rural areas is largely dependent on the public health service. PNS data showed that 35.8% of the men urban population has private health insurance, while 7.5% of the rural population has this service. Therefore, the sample of rural users with private health plans is not sufficient to find possible statistically significant differences between groups (Additional file 1: Table S1).

Given the information presented herein, it is observed that DRE prevalence is still high in the Brazilian population, specially considering the harms involved and lack of conclusive evidence on the benefits of this examination for prostate cancer screening purposes. A study based on prostate, lung, colorectal and ovarian (PLCO) cancer screenings pointed that DRE trial with a normal PSA result led to an additional capture of 2% of men with clinically significant prostate cancer. This incremental gain suggests that DRE screening provides relatively minimal gains, in face of the discomfort and invasive effect of the examinations. Also, DRE seems not to be useful if PSA is going to be dosed anyway. The position of the American Urology Association reinforces that there are no evidences that screening with DRE is beneficial, and affirms that it should not be part of primary screening, but could be potentially useful as secondary examination in men with high PSA levels [35]. Independently of the role of DRE in screening, it presents relevance in clinical practice for several other situations, such as the diagnostic investigation of several diseases – including prostate cancer – and in staging of this type of cancer [36].

A study that verified the geographic pattern and trends for prostate cancer in South and Central America confirmed that the 3–5% increase in the annual incidence of this cancer, along with the maintenance of constant mortality rates between 1997 and 2008, in countries such as Brazil, Argentina, Chile and Costa Rica, was partly due to screening with DRA and/or PSA, and this incidence should continue to increase due to these practices [2]. These data reinforce the existence of increasing overdiagnosis for prostate cancer in Latin America in the most recent decades.

Another factor that may stimulate prostate cancer screening in Brazil is the existence of guidelines that stimulate this practice among the Afro-descendent population. However, there is no high-quality evidence that supports differentiated recommendations for Afro-descendants. The participation of Afro-descendants in PCLO and the European Randomized Study of Screening for Prostate Cancer (ERSPC) clinical trials was very low and no sub-group analysis indicated ethnic-related differences in the results [37]. Moreover, here is a Brazilian study restricted to patients with localized prostate cancer showing worse prognosis for Caucasians than for Afro- descendants [36].

Considering the most recent recommendations for prostate cancer screening in the USA, a study that utilized 2013 data from the Behavioral Risk Factor Surveillance System (BRFSS) with 2248 men aged at least 40 years old indicated that only 36% of men made their decision of screening for prostate cancer in agreement with their medical doctors [38]. In clinical practice, shared decision-making not only is still carried out inconsistently – when practiced – but is accomplished incompletely or wrongfully in function of the unreal expectations of men regarding screening and unpreparedness of most doctors to provide all the necessary information for decision making [39].

Therefore, this shared decision to undergo screening or not constitutes a challenge, both for the private and public sectors. Our results points towards the necessity of redirecting early detection campaigns for prostate cancer, such as blue November, which utilize – generally – the motto of preventing prostate cancer, overestimating the benefits of screening with DRE and omitting the harms involved.

Regarding the limitations of this study, information on screening with DRE is self-reported and therefore is subject to memory and information biases. Also, the results of this type of population-based survey are inclined to underestimate the utilization of DRE, due to discomfort or embarrassment during the self-reporting or overestimate it the interviewees believe they should have undergone DRE [40]. Another limitation is that the history of DRE could be related to the diagnostic investigation of clinical complaints, and not to screening. Due to context of the questions on the survey and the scarcity of these situations in the general population [26], it is considered that its possible impact on the prevalence estimates herein presented is minimal.

## Conclusions

The results of this study demonstrate that prostate cancer screening via DRE is very prevalent in Brazil, and more commonly carried out by private health insurance practionners than by the public health system physicians. The results indicated that, in Brazil, DRE is higher in individuals with low life expectancies (elderly, with worse health perception, with more comorbidities), with healthier lifestyles, and with better access to health services (private health plans, resident of urban zones, with better socioeconomic and education levels). Better access to health services associated with media campaigns promoting screening, probably increased the demands for prostate cancer screening in this group, in addition to the routine offer of opportunistic screening for those that already utilize health services. This medical practice should be influencing the increasing incidence trends for prostate cancer, probably without any impacts on the reduction of prostate cancer mortality. This could also result in substantial overdiagnosis and overtreatment, as has occurred in several high-income countries.

## Supplementary information

**Additional file 1: Table S1.** Proportion (%), respective confidence intervals of private and public healthcare consumers men, according to the demographic and socioeconomic independent.

## Data Availability

The data were obtained from Brazilian Health Survey. They were publicly available from the Brazilian Institute of Geography and Statistics. Available in: https://ww2.ibge.gov.br/home/estatistica/populacao/pns/2013/default_microdados.shtm

## Abbreviations

BRFSS: Behavioral Risk Factor Surveillance System
CCEB: Economic Classification Criterion Brazil
DRE: Digital rectal examination
ERSPC: European Randomized Study of Screening for Prostate Cancer (ERSPC)
IBGE: Brazilian Institute of Geography and Statistics
INCA: Brazilian National Cancer Institute
ISASP: Multi-Center Health Survey of the state of São Paulo
PLCO: Prostate, lung, colorectal and ovarian
PNS: Brazilian National Health Survey
PSA: Prostate-specific antigen
SUS: Brazilian Unified Health System
USPSTF: U.S. Preventive Services Task Force
