# Supplementary material for: Digital rectal examination and its associated factors in the early detection of prostate cancer: a cross-sectional population-based study
Source: BMC Public Health. 2019 Nov 27;19:1573. doi: 10.1186/s12889-019-7946-z (PMC6881979; doi:10.1186/s12889-019-7946-z)
Supplement: Supplementary file 1 — Additional file 1: Table S1. Proportion (%), respective confidence intervals of private and public healthcare consumers men, according to the demographic and socioeconomic independent. [file 12889_2019_7946_MOESM1_ESM.docx]

**Additional file 1: Table S1.** Proportion (%), respective confidence intervals of private and public healthcare consumers men, according to the demographic and socioeconomic independent.

|  |  |  |
| --- | --- | --- |
|  | **Public** | **Private** |
| **Variables** | **Prop (%) CI (95%)** | **Prop (%) CI (95%)** |
|  |  |  |
| **Age group** |  |  |
|  |  |  |
| 50-59 | 67.2 (64.2-70.1) | 32.8 (29.9-35.8) |
| 60-69 | 71.1 (67.6-74.3) | 28.9 (25.7-32.5) |
| 70-79 | 72.0 (67.2-76.3) | 28.0 (23.7-32.9) |
| 80 | 63.5 (54.1-70.3) | 37.5 (29.7-45.9) |
| **Self-reported skin color** | |  |
| White | 60.0 (56.7-63.0) | 41.0 (37.0-43.3) |
| Non white | 78.2(75.6-80.5) | 21.8 (19.5-24.4) |
| **Marital status** |  |  |
| No spouse | 79.0 (75.8-82.0) | 21.0 (18.0-24.2) |
| With spouse | 66.7 (63.2-68.1) | 34.3 (31.9-36.8) |
| **Health self-perception** | | |
| Very good/good | 59.7 (56.7-62.6) | 40.3 (37.4-43.3) |
| Regular | 77.6 (74.5-80.3) | 22.4 (19.7-25.5) |
| Bad/Very bad | 85.4 (80.5-89.4) | 14.6 (10.6-19.7) |
|  |  |  |
| **Brazil Criterion** |  |  |
| A and B | 47.9 (43.1-52.7) | 52.1 (47.3-56.9) |
| C | 71.7 (68.6-74.7) | 28.3 (25.3-31.4) |
| D and E | 78.1 (75.4-80.6) | 21.9 (19.4-24.6) |
|  |  |  |
| **Education level** |  |  |
| Iliterate | 91.4 (88.2-93.7) | 8.64 (6.27-12.3) |
| Primary | 77.4 (75.9-80.7) | 21.6 (19.3-24.1) |
| High school | 54.6 (50.0-59.1) | 45.4 (40.9-50.0) |
| Undergraduate | 19.97 (16.4-23.9) | 80.1 (76.1-83.6) |
|  |  |  |
| **Employment** |  |  |
| Employed | 66.2 (63.4-68.9) | 33.8 (31.1-36.6) |
| Unemployed | 71.8 (68.7-74.8) | 28.2 (25.2-31.3) |
|  |  |  |
| **Geographic region** | |  |
| Southeast | 61.2 (57.4-64.9) | 38.8 (35.1-42.7) |
| South | 63.9 (59.1-68.5) | 36.1 (31.6-40.9) |
| Midwest | 65.6 (61.2-69.7) | 34.4 (30.3-38.8) |
| Northeast | 83.2 (80.4-85.7) | 16.8 (14.3-19.6) |
| North | 82.7 (78.0-86.6) | 17.3 (13.4-22.1) |
| **Housing area** |  |  |
| Urban | 64.2 (61.8-66.6) | 35.8 (33.4-38.2) |
| Rural | 92.5 (90.5-94.1) | 7.50 (5.90-9.46) |
| Source: PNS, 2013. | | |
